# Skeletal muscle heat shock protein 70: diverse functions and therapeutic potential for wasting disorders

**DOI:** 10.3389/fphys.2013.00330

**Published:** 2013-11-11

**Authors:** Sarah M. Senf

**Affiliations:** Department of Physical Therapy, University of FloridaGainesville, FL, USA

**Keywords:** heat shock proteins, muscle atrophy, damage, regeneration, dystrophy, sarcopenia, inflammatory response, muscle dysfunction

## Abstract

The stress-inducible 70-kDa heat shock protein (HSP70) is a highly conserved protein with diverse intracellular and extracellular functions. In skeletal muscle, HSP70 is rapidly induced in response to both non-damaging and damaging stress stimuli including exercise and acute muscle injuries. This upregulation of HSP70 contributes to the maintenance of muscle fiber integrity and facilitates muscle regeneration and recovery. Conversely, HSP70 expression is decreased during muscle inactivity and aging, and evidence supports the loss of HSP70 as a key mechanism which may drive muscle atrophy, contractile dysfunction and reduced regenerative capacity associated with these conditions. To date, the therapeutic benefit of HSP70 upregulation in skeletal muscle has been established in rodent models of muscle injury, muscle atrophy, modified muscle use, aging, and muscular dystrophy, which highlights HSP70 as a key therapeutic target for the treatment of various conditions which negatively affect skeletal muscle mass and function. This article will review these important findings and provide perspective on the unanswered questions related to HSP70 and skeletal muscle plasticity which require further investigation.

## Introduction

The 70 kDa heat shock protein (Hsp70/HSPA) family is one of the most evolutionary conserved protein families across both prokaryotic and eukaryotic organisms (Brocchieri et al., 2008). Due to their key roles as molecular chaperones, members of the Hsp70 family are most widely known for their involvement in promoting cellular proteostasis and survival throughout the lifespan and during periods of stress (Morimoto, 1991). However, this important family of proteins possesses several additional functions, including the regulation of various cell signaling pathways involved in cell growth and inflammation (Asea et al., 2000; Nollen and Morimoto, 2002).

In humans, there are at least 13 different genes that encode for distinct Hsp70 proteins, but which share a common domain structure, including *HSPA1A, HSPA1B, HSPA1L, HSPA2, HSPA4, HSPA4L, HSPA5, HSPA6, HSPA7, HSPA8, HSPA9, HSPA12A*, and *HSPA14* (Kampinga et al., 2009). The protein products encoded by these genes are localized to various subcellular compartments, and differ in their pattern of expression across various tissues. Among the most well studied Hsp70 family members are the constitutively expressed 70 kDa heat shock cognate protein (HSC70 or HSC73), encoded by the *HSPA8* gene, and the stress-inducible Hsp70 family members, encoded for by the *HSPA1A* and *HSPA1B* genes and whose protein products differ by only two amino acids. Due to inconsistencies in nomenclature, the proteins produced by these inducible genes are referred to by several different names. HSP70-1/HSP72/HSPA1/HSPA1A each refers to the protein product of *HSPA1A*, while HSP70-2/HSPA1B both refer to the protein product of *HSPA1B*. Moreover, due to the high homology of HSP70-1 and HSP70-2, and the belief that these are fully interchangeable proteins, these proteins are often collectively referred to as simply HSP70, or more commonly in the muscle literature, as HSP72.

In the last decade, the importance of HSP70 in skeletal muscle has gained significant attention, due to its induction in response to various physiological and pathophysiological stimuli, and its speculated role in the muscle adaptations to these stimuli. This article will therefore, review the studies which have provided key experimental evidence surrounding the biological functions of HSP70 in skeletal muscle. More specifically, this article will focus on the studies which have demonstrated its critical role in (1) protecting against muscle damage, (2) promoting muscle regeneration and recovery, and (3) maintaining skeletal muscle mass and integrity. In doing so, the potential of HSP70-targeted therapeutics for the treatment of both skeletal muscle injuries and muscle wasting disorders will be highlighted. Importantly, since the majority of these studies were performed in rodent models, the use of HSP70 in this article will be in reference to the inducible HSP70 proteins encoded by the rodent *Hspa1a* and *Hspa1b* genes.

## HSP70 and skeletal muscle damage and regeneration

### HSP70 overexpression

The inducible HSP70 is increased in skeletal muscle following various perturbations such as exercise (Hernando and Manso, 1997; Milne and Noble, 2002; Morton et al., 2009) and muscle injury (Senf et al., 2013), and during the periods of muscle re-growth and regeneration associated with these conditions (Selsby et al., 2007; Senf et al., 2013). In contrast, HSP70 is decreased during prolonged periods of muscle inactivity (Lawler et al., 2006; Chen et al., 2007; Senf et al., 2008) when muscles undergo significant remodeling to reduce myofiber size to match the reduced force demands placed on the muscle. Due to these observations, HSP70 has long been considered to play an important role in regulating skeletal muscle plasticity.

The first definitive evidence that HSP70 regulates skeletal muscle plasticity was demonstrated through the use of muscle-specific Hsp70 transgenic (Tg) mice, and was published by McArdle et al. (2004). In this study, muscles from wild type (WT) mice and Hsp70 Tg mice were damaged through inducing lengthening muscle contractions, and the morphological and functional properties of these muscles subsequently compared. The authors found that 3 days following the lengthening contraction protocol, muscles from Hsp70 Tg mice showed less muscle fiber damage compared to WT, and reduced deficits in muscle specific force. At later time points Hsp70 Tg mice also showed earlier muscle functional recovery than WT. Thus, these findings demonstrate that upregulation of HSP70 is sufficient to protect against muscle damage and enhance muscle functional recovery. In a subsequent study, skeletal muscles from Hsp70 Tg mice were also found to have enhanced morphological recovery following muscle injury induced via cryolesioning (Miyabara et al., 2006). This study similarly linked their findings of enhanced recovery in muscles overexpressing HSP70 to reduced muscle damage in response to the injury stimulus. Markers of muscle satellite cell-activation were also measured in injured muscles, and found to be decreased in Hsp70 Tg mice compared to WT. This finding was not surprising since the extent of satellite cell-mediated repair should match the extent of muscle damage. In a more recent study, muscles from Hsp70 Tg mice also displayed enhanced recovery of fiber cross-sectional area (CSA) and function following a period of disuse muscle atrophy (Miyabara et al., 2012). Thus, these studies together provide considerable evidence that HSP70 upregulation is a feasible countermeasure to protect against muscle damage and enhance the recovery process.

A later study by Moresi et al. (2009) further demonstrated a more specific role of HSP70 in the augmentation of muscle fiber regeneration. In this study, HSP70 was overexpressed specifically in regenerating myofibers via electroporation of an *Hsp70* expression plasmid into muscles 3 days *following* cryolesioning injury. Due to the timing of Hsp70 overexpression in muscles *post-injury*, this therefore, bypassed and eliminated the potential for ectopic HSP70 to interfere with the early damage sequela. When the CSA of regenerating myofibers positive for HSP70 or a control vector were measured and compared in muscles 7 days *post-injury* (4 days following plasmid electroporation), regenerating myofibers positive for HSP70 were significantly larger. Thus, the data from this study provided clear evidence that enhancing HSP70 expression *post-injury* can enhance the muscle regenerative process. This is highly significant from a therapeutic standpoint, since preventative treatments to reduce muscle damage in the event of acute injury are often unrealistic. Thus, HSP70-targeted therapeutics to enhance muscle regeneration and recovery may be particularly relevant to sports-related skeletal muscle strains and contusions, traumatic muscle injures and even spinal cord injury (SCI), since markers of regeneration were recently shown to be elevated in rat skeletal muscle following moderate spinal cord contusion (Jayaraman et al., 2013).

### HSP70 knockout

Collectively the studies discussed thus, far have provided key experimental evidence that Hsp70 upregulation protects against muscle damage and enhances the recovery process. However, until just recently, it was not clear whether HSP70 is necessary for the regenerative process, and whether a reduction in HSP70 alone is sufficient to enhance muscle damage and interfere with the regenerative process. This question is important in that skeletal muscle expression of HSP70, and the ability to induce its expression, is diminished with age concomitantly with the age-related decline in muscle regenerative capacity and rate of recovery following muscle damage (Vasilaki et al., 2002; McArdle et al., 2004). The study by McArdle et al. (2004) provided important evidence that these two events may be linked, since both adult and aged mice overexpressing HSP70 showed protection against muscle damage and enhanced functional recovery. However, loss of function studies using gene knockdown are also important in confirming this link, through establishing the biological *requirement* of HSP70 for these skeletal muscle processes. Therefore, my colleagues and I recently conducted experiments using mice which lack the inducible HSP70 coded for by *Hspa1a* and *Hspa1b (Hsp70^−/−^* mice). In these experiments muscles from WT and *Hsp70^−/−^* mice were injured via direct injection with cardiotoxin, which induces widespread muscle fiber necrosis, and is thus, a standardized and reproducible method to study the regenerative process. At various time points following the injury stimulus the extent of muscle inflammation, necrosis, and regeneration were subsequently compared. We found that injured muscles from *Hsp70^−/−^* mice had a significantly delayed inflammatory response to muscle injury which was followed at later time points by sustained inflammation and muscle fiber necrosis, fibrosis, and reduced CSA of regenerating myofibers (Senf et al., 2013). In addition, injured muscles lacking HSP70 also developed widespread calcifications during the recovery process. These findings therefore, provide strong evidence that HSP70 is necessary for successful muscle regeneration and recovery, and further link the age-related impairments in these critical skeletal muscle processes to deficits in HSP70.

The mechanisms whereby HSP70 regulates muscle regeneration and recovery following injury are still being elucidated. However, in our recent report we uncovered some important mechanistic details. Based on rescue experiments introducing an *Hsp70* plasmid into muscles of *Hsp70^−/−^* mice either 4 days prior to injury or 4 days post injury, we establish that HSP70 plays an especially critical role within the first 4 days following injury to support the recovery process (Senf et al., 2013). The first several days following muscle injury are dominated by a highly coordinated inflammatory response involving the recruitment of various immune cell populations including neutrophils and macrophages which support muscle regeneration and repair (Tidball and Villalta, 2010). Infiltration of these immune cell populations contribute to muscle healing in various capacities, and their failure to infiltrate in a timely manner can certainly impede the recovery process. One important role is their involvement in the removal of necrotic cellular debris through phagocytosis, which allows viable muscle cells to successfully repopulate damaged areas. Since injured muscles from *Hsp70^−/−^* mice displayed a significantly impaired ability to recruit these important phagocytic immune cells in a timely manner, we hypothesize that this may have contributed to the persisting muscle inflammation and fiber necrosis seen in these mice at later time points. This notion is supported by the rescue experiments in which reintroduction of HSP70 into muscles of *Hsp70^−/−^* mice *prior to* cardiotoxin injury (but not post injury) prevented these deficits in muscle recovery.

So exactly how does HSP70 expressed by skeletal muscle fibers contribute to immune cell infiltration in response to injury? One possibility is through the immunostimulatory functions of HSP70 when localized to the extracellular environment. Studies on tissue injury of the heart, liver and skin have in fact demonstrated that HSP70 is released into the extracellular environment following tissue injury and facilitates the activation of pro-inflammatory processes and the recruitment of immune cells to the injury site (Dybdahl et al., 2002, 2005; Kimura et al., 2004; Kovalchin et al., 2006). Thus, a similar mechanism involving extracellular HSP70 may be involved in the inflammatory response to muscle injury. While this concept has previously been suggested by others (Lightfoot et al., 2009; Han et al., 2010), data generated in our recent study were the first to support this notion. In these experiments, recombinant HSP70 protein was directly injected into muscles of *Hsp70^−/−^* mice at the time of injury to simulate HSP70 release into the extracellular microenvironment. Using this method, we were able to completely restore early immune cell infiltration into injured muscles of *Hsp70^−/−^* mice, thus, providing a novel link between the inflammatory response to muscle injury and the extracellular functions of HSP70 (Senf et al., 2013). However, our understanding of HSP70 and its involvement in the inflammatory response to muscle injury is still in its infancy. Thus, additional studies are needed to elucidate the precise roles of HSP70 in the initiation of inflammatory processes in damaged muscle, and how this modulates the regenerative process.

As mentioned previously, the CSA of regenerating myofibers in muscles from *Hsp70^−/−^* mice were significantly smaller than WT (Senf et al., 2013). This indicates that HSP70 is necessary for the normal muscle fiber regeneration, and complements the previous findings of Moresi et al. (2009) which demonstrated that HSP70 overexpression enhances the CSA of regenerating myofibers. Although the mechanisms whereby HSP70 is necessary for myofiber regeneration are currently unclear, markers of satellite cell activation and proliferation in injured muscles from *Hsp70^−/−^* mice were not significantly compromised. Therefore, HSP70 may be dispensable for these early stages of the myogenic program. In contrast, HSP70 could regulate later stages of the myogenic program which support differentiation and the formation of multinucleated myofibers. This notion is supported by experiments from two separate studies in which introduction of an *Hsp70* plasmid into muscles 3 or 4 days following injury enhanced the CSA and nucleation of regenerating myofibers (Moresi et al., 2009; Senf et al., 2013). However, as mentioned previously, additional studies detailing the effect of HSP70 overexpression and knockdown on each stage of the myogenic program are needed to better understand the role of HSP70 in these important cellular processes. Preferably, these experiments would be performed in both skeletal muscle cells *in vitro* and whole muscle, *in vivo*, to differentiate between the muscle cell autonomous and non-autonomous mechanisms whereby HSP70 regulates the muscle regenerative process following injury.

## HSP70 and muscle wasting and dysfunction

In addition to the roles of HSP70 in facilitating muscle regeneration and recovery following injury, HSP70 also plays an important role in regulating muscle fiber size under baseline conditions and during conditions of muscle atrophy. Indeed, HSP70 is decreased in both adult and aged rats during periods of muscle disuse, and plasmid-mediated restoration of HSP70 expression in muscles during the disuse period significantly inhibits the associated fiber atrophy (Senf et al., 2008; Dodd et al., 2009). These findings were linked to HSP70 negatively regulating the Forkhead BoxO (FoxO) and Nuclear Factor κ B (NF-κ B) pathways, which are activated during multiple conditions of muscle atrophy and which drive the atrophy phenotype (Cai et al., 2004; Hunter and Kandarian, 2004; Sandri et al., 2004; Judge et al., 2007; Senf et al., 2010; Reed et al., 2012). Thus, reductions in HSP70 during conditions of atrophy may contribute to the atrophy phenotype through weakening the inhibition of these signaling pathways. Evidence that HSP70 is necessary for the maintenance of muscle fiber size and functional integrity is demonstrated by the muscle phenotype of adult *Hsp70^−/−^* mice, in which muscle fiber CSA and muscle specific force is reduced when compared to controls (Senf et al., 2013). This is further supported by evidence that the age-related decline in muscle specific force is prevented in muscles of aged mice overexpressing HSP70 throughout the lifespan (McArdle et al., 2004). This regulation of skeletal muscle functional integrity by HSP70 may be related to the finding that muscles from Hsp70 Tg mice also displayed enhanced antioxidant capacity (Broome et al., 2006), since oxidative stress may contribute to muscle dysfunction during the aging process. In addition, and perhaps related to this, HSP70 also appears to contribute to the maintenance of skeletal muscle quality during normal physiological conditions, since muscles from *Hsp70^−/−^* mice also had significant increases in the amount of extracellular tissue (non-muscle fiber tissue) surrounding the already smaller muscle fibers. The mechanisms responsible for the reduced fiber CSA in mice lacking HSP70 throughout the lifespan are not known. However, since induction of intracellular HSP70 represses pathways involved in muscle atrophy and inflammation in skeletal muscle (Chung et al., 2008; Senf et al., 2008), the lack of HSP70 may contribute to increases in these signaling pathways under baseline conditions. Alternatively, deficits in post-natal muscle growth in mice could play a role in this finding, since *Hsp70^−/−^* mice had deficits in the growth of regenerating myofibers, and both processes rely upon related cellular mechanisms to support fiber growth. Clearly, numerous unanswered questions still remain surrounding the mechanisms in which HSP70 regulates muscle fiber size and function. Nonetheless, these collective studies confirm that HSP70 is necessary for the maintenance of muscle fiber size and functional integrity, and highlight HSP70 upregulation as a key therapeutic strategy that may be beneficial during various skeletal muscle wasting disorders to maintain muscle mass and function.

One highly significant study surrounding the use of HSP70-targeted therapeutics for genetic muscle wasting disorders was recently published by Gehrig et al. (2012). In this study, the authors demonstrate that upregulation of the inducible HSP70 in mouse skeletal muscle (through either genetic or pharmacological means) ameliorates the dystrophic phenotype. This finding was demonstrated in two models of muscular dystrophy related to the absence of dystrophin, including the mdx model and the more severe dko model in which utrophin is also absent. Importantly, treatment of dystrophic mice with BGP-15, a pharmacological co-inducer of HSP70 that is currently being used in clinical trials, improved the dystrophic pathology of both limb and diaphragm muscles and extended lifespan. Thus, BGP-15 and other pharmacological inducers of HSP70 may be key therapeutic agents for muscular dystrophies. The mechanism whereby HSP70 improved the dystrophy phenotype in this study was linked to its regulation of the sarcoplasmic/endoplasmic reticulum Ca(2+)-ATPase (SERCA) protein, whose activity is compromised in severely dystrophic muscle. The activity of SERCA is critical in removing intracellular calcium, and HSP70 was found to interact with and enhance SERCA activity. Since increased intracellular calcium is a key activator of inflammatory and muscle degenerative pathways, maintenance of SERCA activity was proposed to be a key mechanism for the amelioration of the dystrophic phenotype in muscles overexpressing HSP70. However, since HSP70 also negatively regulates muscle atrophy pathways, enhances regenerative processes and promotes a timely and controlled inflammatory response to muscle damage, the beneficial effects of HSP70 upregulation on the dystrophic pathology could also be related to these additional skeletal muscle functions of HSP70. Regardless of the mechanism, the findings from this study clearly indicate that HSP70-targeted therapeutics have significant potential for the treatment of dystrophic muscle pathologies.

## Conclusion

In summary, the inducible HSP70 is a critical skeletal muscle protein that positively regulates muscle size and function during health and disease. The studies which directly support this notion are summarized in Table 1. While several questions still remain surrounding the mechanisms responsible for this regulation, it is clear that therapeutics targeting HSP70 upregulation have strong potential for success in the treatment of both acquired and genetic muscle wasting disorders and in the treatment of muscle injuries. However, future studies should continue to delineate the cellular mechanisms whereby HSP70 regulates skeletal muscle plasticity. These studies are important not only from a mechanistic standpoint, but from a therapeutic standpoint, since the timing of HSP70 induction, route of administration, and duration of treatment to optimally enhance therapeutic benefits may be revealed from knowledge gained through these studies.

**Table 1:** **List of studies which have directly manipulated Hsp70 expression to investigate the functions of Hsp70 in regulating skeletal muscle plasticity**.

	**Condition/model**	**Skeletal muscle phenotype**	**References**
**Hsp70 OVEREXPRESSION**			
Hsp70 transgenic mice	10–12 months adult mice	Decreased body and muscle mass	McArdle et al., 2004
	Aging	Preservation of muscle specific force	McArdle et al., 2004
	Muscle damage/eccentric contractions	Decreased muscle damage, improved muscle functional recovery (adult and old mice)	McArdle et al., 2004
	Muscle damage/cryolesioning	Decreased muscle damage, improved morphological recovery	Miyabara et al., 2006
	Modified muscle use/cast- immobilization and recovery	Improved muscle structural and functional recovery	Miyabara et al., 2012
	Muscular dystrophy/crossed with *mdx* mice	Improved muscle morphology and function	Gehrig et al., 2012
Hsp70 plasmid electroporation	Muscle atrophy/cast-immobilization	Attenuation of disuse muscle fiber atrophy (adult and old rats)	Senf et al., 2008; Dodd et al., 2009
	Muscle damage/freeze injury	Increased CSA of regenerating muscle fibers	Moresi et al., 2009
**Hsp70 KNOCKDOWN**			
Hsp70 knockout mice	12–15 weeks adult mice	Decreased muscle fiber CSA, decreased muscle specific force	Senf et al., 2013
	Muscle damage/direct cardiotoxin injection	Delayed inflammatory response to injury, impaired muscle regeneration and recovery (smaller CSA of regenerating fibers, sustained inflammation, fibrosis and calcium deposition)	Senf et al., 2013
	Modified muscle use/cast-immobilization and recovery	Decreased muscle reloading damage, decreased size of regenerating fibers	Senf et al., 2013

### Conflict of interest statement

The author declares that the research was conducted in the absence of any commercial or financial relationships that could be construed as a potential conflict of interest.
